# Supplementary material for: Cocktail, a Computer Program for Modelling Bacteriophage Infection Kinetics
Source: Viruses. 2022 Nov 9;14(11):2483. doi: 10.3390/v14112483 (PMC9695944; doi:10.3390/v14112483)
Supplement: Supplementary file 1 [file viruses-14-02483-s001.zip › Supplementary material/Cocktail.pdf]

The Cocktail program

The purpose of the Cocktail program is to model the infection dynamics of one, or a combination of two, phage(s) infecting a bacterial species growing in a vessel in a constant volume of nutrient and under varying relevant parameter settings. The aim is to supply an easier way to carry out modelling in phage infection biology, for a better understanding of the complex dynamics during phage therapy. The underlying mathematical models are described in Nilsson AS: “Cocktail. A computer program for modelling bacteriophage infection kinetics” to be published in *Viruses* https://www.mdpi.com/journal/viruses. It is also a good idea to read the previously published articles by Levin *et al.* [1], Lenski [2], Levin and Bull [3], Gill [4] and Abedon [5]. The following information describes the models and model settings in brief.

Bacteria can grow at a rate, ψ, based on a function dependent on available nutrients, *C*, bacteria requirement for dividing, ε and a constant, *K*. Bacteria can also flow out of the system at a rate of ω, decay or being neutralised at a rate of γ, and be resistant to phages either from the start at a certain frequency or become resistant during the run at a rate of μ. Resistant bacteria can be better or less fit and have different growth rates, ψ_{RA}, ψ_{RB}, ψ_{RAB}. Parameter default values and allowed value ranges are indicated in the table below.

Two phages, *A* and *B*, with different characteristics can be added to the system at three different timepoints. The phages may differ in their latent periods, *l*, in their capacity to bind to the bacteria at an adsorption rate of δ, and by the burst size, the number of phages produced per cell β. Phages can also flow out of the system at a rate of ω and decay at a rate of φ.

Three models can be applied for phage adsorption, mutations and bacteria moving into a refuge. Primary phage adsorption can be either set as “Standard” as in most mathematical models or “Poisson”. With the “Standard” setting, bacteria are adsorbed by one phage per time step whereas in the “Poisson” setting bacteria adsorb according to a Poisson probability with a mean of phages/bacteria (sometimes referred to as MOI_{actual} [6]). With the secondary adsorption “Uninfected” set, phages adsorb to uninfected cells only but with the “Susceptible” option set, phages adsorb to infected bacteria as well [5].

Mutations to resistance against one or both phages can be set to either “Deterministic” or “Stochastic”. In the “Deterministic” mode bacteria resistant to phages are introduced at the chosen mutation rate × the number of newly divided bacteria. The “Stochastic” option introduces resistant cells randomly according to Poisson probabilities with the same mean. If the mean equals or is above 10, the number of added resistant cells are randomised from a normal distribution.

In the “Refuge cells” setting, bacteria are metabolically inactive either in a “Planktonic” state or in a layered protected state, “LIFO” (the last cells to enter the refuge are first out). They are exempted from phage infection at a rate of σ in these states but can become susceptible to phage infection again at a rate of ρ. In the model setting “Standard” and with the option “Susceptible” set however, bacteria adsorbs phages but do not get infected.

The discretisation error can be minimised at the expense of the running time by setting the “Time step size” to a lower value.

Source codes and updates can be found at GitHub:
https://github.com/ASNilsson/Cocktail-phage-infection-kinetics

Parameter settings:

Symbol	Description	Start values Default	Allowed range	Unit
Bacteria				
S	Uninfected, susceptible bacteria	1×10^5	$10\text{-}1\times10^{12}$	CFU/ml
I_A	Bacteria infected by phage *A*	-	-	|
I_B	Bacteria infected by phage *B*	-	-	|
I_{AB}	Bacteria infected by phages *A* and *B*	-	-	|
R_A	Bacteria resistant to phage *A*	1×10^{-7}	$0\text{-}1\times10^{-2}$	|
R_B	Bacteria resistant to phage *B*	1×10^{-7}	$0\text{-}1\times10^{-2}$	|
R_{AB}	Bacteria resistant to phages *A* and *B*	1×10^{-14}	$0\text{-}1\times10^{-6}$	|
R_{AIB}	Bacteria resistant to *A* infected with *B*	-	-	|
R_{BIA}	Bacteria resistant to *B* infected with *A*	-	-	|
S_r	Susceptible bacteria in a refuge	0	-	|
R_{rA}	Bacteria resistant to *A* in a refuge	-	-	|
R_{rB}	Bacteria resistant to *B* in a refuge	-	-	|
R_{rAB}	Bacteria resistant to *AB* in a refuge	-	-	CFU/ml
Parameters				
ψ	Growth rate of *S*	0.7	0-1.5	/h
K	Monod constant	5.0	0.01-100	µg/ml*
ε	Resource for division of one bacterium	2×10^{-6}	$1\times10^{-8}\text{-}1\times10^{-4}$	µg/cell*
γ	Bacterial decay rate	0	0-1	/h
μ_A	Mutation rate for resistance against *A*	1×10^{-7}	$0\text{-}1\times10^{-4}$	/cell div.
μ_B	Mutation rate for resistance against *B*	1×10^{-7}	$0\text{-}1\times10^{-4}$	/cell div.
ψ_{R_A}	Growth rate of R_A	0.7	0-1.5	/h
ψ_{R_B}	Growth rate of R_B	0.7	0-1.5	/h
$\psi_{R_{AB}}$	Growth rate of R_{AB}	0.7	0-1.5	/h
σ	Rate of bacteria into refuge	0	0-0.01	/min
ρ	Rate of bacteria out from refuge	0	0-0.01	/min
C_0	Available resources from start	100	0-1000	µg/ml*
C	Resources flowing in from a reservoir	100	0-1000	µg/ml*
ω	Flow rate	0.2	0-100	/h
Phages				
Parameters				
A	Titre of phage *A*	1×10^8	$0\text{-}1\times10^{13}$	PFU/ml
B	Titre of phage *B*	1×10^8	$0\text{-}1\times10^{13}$	PFU/ml
δ_A	Adsorption rate of *A*	1×10^{-10}	1×10^{-14} - 1×10^{-7}	ml/min
δ_B	Adsorption rate of *B*	1×10^{-10}	1×10^{-14} - 1×10^{-7}	ml/min
l_A	Latent period of *A*	30	1-60	min
l_B	Latent period of *B*	20	1-60	min
β_A	Burst size of *A*	100	0-1000	PFU/cell
β_B	Burst size of *B*	100	0-1000	PFU/cell
φ_A	Decay rate of phage *A*	0	0-1	/h
φ_B	Decay rate of phage *B*	0	0-1	/h

* The symbol for the micro prefix, "μ", is denoted by "u" in the program user interface.

Input formats

Values can in general be entered with three significant digits. If the input should be an integer it can be given either as that or in scientific notation. Real numbers should be given either in decimal or scientific format with a point as the decimal separator.

Integers: e.g. 100000000, 1.0E+8, 1.67E+8, 1E+8 or 1E8
Real numbers: e.g. 0.00000001, 1.0E−8, 1.76E−8 or 1E−8

License and citation

The Cocktail program and source code files are distributed under the license Creative Commons Attribution-NonCommercial-ShareAlike 4.0 International License. In short, this means that it is free for everyone to use, to modify the source code, build upon the program or code, and free to distribute in any medium. You must however give appropriate credit and a link to the license. If changes were made to the program or code, these must be specified, and distribution of modifications must be under the same license. It is not allowed for anyone to use any part of the program or code for commercial purposes. A short description of the license can be found at: https://creativecommons.org/licenses/by-nc-sa/4.0/. The license and program version number can be found by double clicking anywhere in the Cocktail parameter settings window.
A more thorough review of the Cocktail program, and the proper article to cite, can be found in: Nilsson AS. 2022. Cocktail, a computer program for modelling bacteriophage infection kinetics. To be published in Viruses at https://www.mdpi.com/journal/viruses/special_issues/Bacteriophage_Nordic.

References

1. Levin, B.R.; Stewart, F.M.; Chao, L. Resource-Limited Growth, Competition, and Predation: A Model and Experimental Studies with Bacteria and Bacteriophage. *Am. Nat.* **1977**, *111*, 23.
2. Lenski, R.E. Dynamics of interactions between bacteria and virulent bacteriophage. *Adv. Microb. Ecol.* **1988**, *10*, 1–44.
3. Levin, B.R.; Bull, J.J. Population and evolutionary dynamics of phage therapy. *Nat Rev Microbiol* **2004**, *2*, 166–173.
4. Gill, J.J. Modeling of bacteriophage therapy. In *Bacteriophage Ecology: Population Growth, Evolution, and Impact of Bacterial Viruses*; Abedon, S.T., Ed.; Cambridge University Press, **2008**; pp. 439–464 ISBN 9780511541483.
5. Abedon, S. Deconstructing Chemostats Towards Greater Phage-Modeling Precision. In *Contemporary Trends in Bacteriophage Research*; Adams, H., Ed.; Nova Science Publishers, **2009**; pp. 249–283 ISBN 9781606921814.
6. Kasman, L.M.; Kasman, A.; Westwater, C.; Dolan, J.; Schmidt, M.G.; Norris, J.S. Overcoming the phage replication threshold: a mathematical model with implications for phage therapy. *J Virol* **2002**, *76*, 5557–5564.
