# Supplementary material for: Cocktail, a Computer Program for Modelling Bacteriophage Infection Kinetics
Source: Viruses. 2022 Nov 9;14(11):2483. doi: 10.3390/v14112483 (PMC9695944; doi:10.3390/v14112483)
Supplement: Supplementary file 1 [file viruses-14-02483-s001.zip › Supplementary material/Tables/Table 1.pdf]

Table S1. Symbols and parameters

		Start values		
Symbol	Description	Default	Allowed range	Unit
Bacteria				
S	Uninfected, susceptible bacteria	1×10^{5}	$10\text{-}1\times10^{12}$	CFU/ml
I_A	Bacteria infected by phage *A*	-	-	|
I_B	Bacteria infected by phage *B*	-	-	|
I_{AB}	Bacteria infected by phages *A* and *B*	-	-	|
R_A	Bacteria resistant to phage *A*	1×10^{-7}	$0\text{-}1\times10^{-2}$	|
R_B	Bacteria resistant to phage *B*	1×10^{-7}	$0\text{-}1\times10^{-2}$	|
R_{AB}	Bacteria resistant to phages *A* and *B*	1×10^{-14}	$0\text{-}1\times10^{-6}$	|
R_{AIB}	Bacteria resistant to *A* infected with *B*	-	-	|
R_{BIA}	Bacteria resistant to *B* infected with *A*	-	-	|
S_r	Susceptible bacteria in a refuge	0	-	|
R_{rA}	Bacteria resistant to *A* in a refuge	-	-	|
R_{rB}	Bacteria resistant to *B* in a refuge	-	-	|
R_{rAB}	Bacteria resistant to *AB* in a refuge	-	-	CFU/ml
Parameters				
ψ	Growth rate of *S*	0.7	0-1.5	/h
K	Monod constant	5.0	0.01-100	µg/ml*
ε	Resource for division of one bacterium	2×10^{-6}	$1\times10^{-8}\text{-}1\times10^{-4}$	µg/cell*
γ	Bacterial decay rate	0	0-1	/h
μ_A	Mutation rate for resistance against *A*	1×10^{-7}	$0\text{-}1\times10^{-2}$	/cell div.
μ_B	Mutation rate for resistance against *B*	1×10^{-7}	$0\text{-}1\times10^{-2}$	/cell div.
ψ_{R_A}	Growth rate of R_A	0.7	0-1.5	/h
ψ_{R_B}	Growth rate of R_B	0.7	0-1.5	/h
$\psi_{R_{AB}}$	Growth rate of R_{AB}	0.7	0-1.5	/h
σ	Rate of bacteria into refuge	0	0-0.01	/min
ρ	Rate of bacteria out from refuge	0	0-0.01	/min
C_0	Available resources from start	100	0-1000	µg/ml*
C	Resources flowing in from a reservoir	100	0-1000	µg/ml*
ω	Flow rate	0.2	0-100	/h
Phages				
Parameters				
A	Titre of phage *A*	1×10^{8}	$0\text{-}1\times10^{13}$	PFU/ml
B	Titre of phage *B*	1×10^{8}	$0\text{-}1\times10^{13}$	PFU/ml
δ_A	Adsorption rate of *A*	1×10^{-10}	$1\times10^{-14}\text{-}\ 1\times10^{-7}$	ml/min
δ_B	Adsorption rate of *B*	1×10^{-10}	$1\times10^{-14}\text{-}\ 1\times10^{-7}$	ml/min
l_A	Latent period of *A*	30	1-60	min
l_B	Latent period of *B*	20	1-60	min
β_A	Burst size of *A*	100	0-1000	PFU/cell
β_B	Burst size of *B*	100	0-1000	PFU/cell
φ_A	Decay rate of phage *A*	0	0-1	/h
φ_B	Decay rate of phage *B*	0	0-1	/h

* The symbol for the micro prefix, "μ", is denoted "u" in the program user interface.
