# Supplementary material for: Cocktail, a Computer Program for Modelling Bacteriophage Infection Kinetics
Source: Viruses. 2022 Nov 9;14(11):2483. doi: 10.3390/v14112483 (PMC9695944; doi:10.3390/v14112483)
Supplement: Supplementary file 1 [file viruses-14-02483-s001.zip › Supplementary material/Tables/Table 2.pdf]

Table S2. Adsorbed *A* and *B* phages per unit time under the Primary and Secondary adsorption settings.

Primary adsorption setting	Standard		Poisson	
Secondary adsorption setting	Uninfected	Susceptible	Uninfected	Susceptible
	Phages adsorb one at a time to uninfected non-resistant cells	Phages adsorb one at a time to non-resistant cells	A number of phages adsorb according to a Poisson probability with lambda = MOI to uninfected non-resistant cells	A number of phages adsorb according to a Poisson probability with lambda = MOI to non-resistant cells
Bacteria	Conceivably adsorbing phages			
S = Susceptible	*A* or *B*	*A* or *B*	*A* and *B*	*A* and *B*
I_A = Infected by *A*	*B*	*A* or *B*	*B*	*A* and *B*
I_B = Infected by *B*	*A*	*A* or *B*	*A*	*A* and *B*
I_{AB} = Infected by *A* and *B*	-	*A* or *B*	-	*A* and *B*
R_A = Resistant to infections by *A*	*B*	*B*	*B*	*B*
R_B = Resistant to infections by *B*	*A*	*A*	*A*	*A*
R_{AB} = Resistant to infections by *A* and *B*	-	-	-	-
R_{AIB} = Resistant to infections by *A* infected with *B*	-	*B*	-	*B*
R_{BIA} = Resistant to infections by *B* infected with *A*	-	*A*	-	*A*
S_r = Susceptible planktonic bacteria in a refuge	-	*A* or *B*<br>No infection	*A* and *B*<br>No infection	*A* and *B*<br>No infection
R_{rA} = Planktonic bacteria resistant to *A* in a refuge	-	*B*<br>No infection	*B*<br>No infection	*B*<br>No infection
R_{rB} = Planktonic bacteria resistant to *B* in a refuge	-	*A*<br>No infection	*A*<br>No infection	*A*<br>No infection
R_{rAB} = Planktonic bacteria resistant to *AB* in a refuge	-	-	-	-
S_r = Susceptible bacteria in a LIFO refuge	-	-	-	-

R_{rA} = Bacteria resistant to *A* in a LIFO refuge	-	-	-	-
R_{rB} = Bacteria resistant to *B* in a LIFO refuge	-	-	-	-
R_{rAB} = Bacteria resistant to *AB* in a LIFO refuge	-	-	-	-
